# Sour Milk in Rheumatoid Arthritis

**Published:** 1909-01-23

**Authors:** Frederick W. Collingwood

**Affiliations:** "Denehurst," Acton Hill, W.


### Editor's Letter-Box.

### SOUR MILK IN RHEUMATOID ARTHRITIS.

Sir,—In respect to notes on soured milk (in your paper) given in cases of rheumatoid arthritis, etc., I should like to mention the case of an old lady over eighty years of age who was suffering from flatulence, senile decay, and rheumatoid arthritis. The soured milk was primarily given for the digestive troubles, which are certainly improved; but the most noticeable fact is that the faint troubles after taking the soured milk for nine weeks are much improved, there is less stiffness, and the pain is almost absent. I think this case may be of interest.

Yours faithfully,

Frederick W. Collingwood.

"Denehurst," Acton Hill, W., January 17, 1909.

